# Consistent physiological, ecological and evolutionary effects of fire regime on conservative leaf economics strategies in plant communities

**DOI:** 10.1111/ele.14182

**Published:** 2023-02-23

**Authors:** Adam F. A. Pellegrini, Leander Anderegg, Jesús N. Pinto‐Ledezma, Jeannine Cavender‐Bares, Sarah E. Hobbie, Peter B. Reich

**Affiliations:** ^1^ Department of Plant Sciences University of Cambridge Cambridge UK; ^2^ Institute for Global Change Biology and School for Environment and Sustainability University of Michigan Ann Arbor Michigan USA; ^3^ Department of Ecology, Evolution, and Marine Biology University of California Santa Barbara California USA; ^4^ Department of Ecology, Evolution, and Behavior University of Minnesota St. Paul Minnesota USA; ^5^ Department of Forest Resources University of Minnesota St. Paul Minnesota USA; ^6^ Hawkesbury Institute for the Environment Western Sydney University Penrith New South Wales Australia

**Keywords:** eco‐evolution, functional traits, leaf physiology, nutrient cycling, phylogenetic constraints, savanna, stoichiometry

## Abstract

The functional response of plant communities to disturbance is hypothesised to be controlled by changes in environmental conditions and evolutionary history of species within the community. However, separating these influences using direct manipulations of repeated disturbances within ecosystems is rare. We evaluated how 41 years of manipulated fire affected plant leaf economics by sampling 89 plant species across a savanna‐forest ecotone. Greater fire frequencies created a high‐light and low‐nitrogen environment, with more diverse communities that contained denser leaves and lower foliar nitrogen content. Strong trait–fire coupling resulted from the combination of significant intraspecific trait–fire correlations being in the same direction as interspecific trait differences arising through the turnover in functional composition along the fire‐frequency gradient. Turnover among specific clades helped explain trait–fire trends, but traits were relatively labile. Overall, repeated burning led to reinforcing selective pressures that produced diverse plant communities dominated by conservative resource‐use strategies and slow soil nitrogen cycling.

## Graphical abstract

Forty‐one years of repeated burning led to reinforcing selective pressures that produced diverse plant communities dominated by conservative resource‐use strategies and slow soil nitrogen cycling.

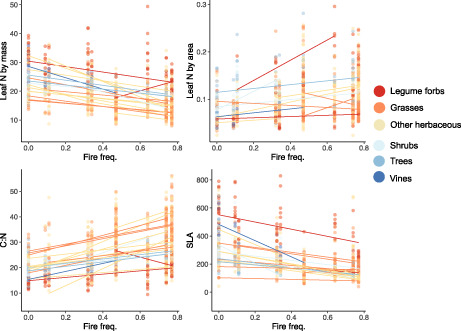

## PEER REVIEW

The peer review history for this article is available at https://publons.com/publon/10.1111/ele.14182.

## OPEN RESEARCH BADGES

This article has earned Open Data and Open Materials badges. Data and materials are available at: https://doi.org/10.6084/m9.figshare.21572373.

## INTRODUCTION

Traits related to resource use and acquisition are hypothesised to predict the occurrence of species in disturbed environments because they can determine plant growth after a disturbance (Cavender‐Bares & Reich, 2012; Odum, 1969; Poorter et al., 2004; Tilman, 1985). In theory, plants optimise their traits according to available resources, which should result in predictable changes in plant traits across environmental gradients (Aerts & Chapin, 1999; Hobbie, 2015). The changes in traits can emerge due to changes in the presence and abundance of species with different traits and/or intraspecific variation in traits across the gradient. Thus, the functional traits of plant communities and species should track disturbance‐driven changes in resource availability, with lower resource availability selecting for conservative strategies and more higher resource availability selecting for acquisitive strategies (Cavender‐Bares & Reich, 2012; Poorter & Bongers, 2006; Reich et al., 1995).

However, predictable shifts in traits following increased disturbance frequency and/or intensity are not always observed (Cavender‐Bares & Reich, 2012; Pellegrini et al., 2021; Reich et al., 1995). The broader literature on plant traits proposes several processes that mediate the magnitude of trait responses to environmental gradients: (i) the magnitude of environmental change determines the magnitude of trait changes, (ii) the plant lineages present are constrained in their functional variation, and (iii) physiological trade‐offs between traits that constrain the ability to optimise to new environmental conditions (Ackerly, 2004; Ackerly et al., 2000; Cavender‐Bares et al., 2004; Cornwell & Ackerly, 2009).

Here, we tested how repeated disturbance by fire changed plant traits from individual to community scales. We focused on the traits (1) specific leaf area (SLA, cm^2^ leaf area per g dry mass), which is a function of leaf thickness and density and an indicator of relative investment in light capture at the expense of physical and physiological leaf robustness, and (2) leaf nitrogen per unit mass (N, %), which is often correlated with leaf photosynthetic capacity and is an indicator of how conservatively a plant uses N (Reich, 2014; Reich et al., 1997; Wright et al., 2004). Together these are important (although by no means the only) traits relevant to the inverse gradient in light and N availability we focused on in our study. Both traits are central components of the leaf economics spectrum (LES) (Reich et al., 1997; Wright et al., 2004), and among species tend to be positively correlated along a spectrum from ‘slow’ resource‐conservative traits (low‐SLA and low‐N leaves with long lifespans) to ‘fast’ resource‐acquisitive traits (high‐SLA and high‐N leaves with short lifespans) (Reich, 2014). However, the strength of the LES (i.e. coupling between traits) can depend on the scale of the comparison, with weaker trait correlations at finer scales (e.g. within‐species) (Anderegg et al., 2018; Lusk et al., 2008). If species' traits change along an environmental gradient in the opposite direction as traits change due to turnover in community composition (i.e. cover of different functional types), trait–environment relationships at the community level can be weak (Ackerly & Cornwell, 2007; Messier et al., 2017).

Fire in savannas has two key effects on plant resources—increasing light availability by reducing tree cover and decreasing N availability via N volatilisation during combustion—that could change plant SLA and leaf N (Dantas et al., 2013; Hoffmann et al., 2012; Pellegrini et al., 2018, 2021; Reich et al., 2001). However, trait responses to fire that are driven by species turnover and/or replacement versus those driven by within‐species responses such as phenotypic plasticity may either reinforce or counteract each other. For example, leaf N per unit mass should increase with soil N availability both across and within species, leading to increases in leaf N with decreased fire frequency and subsequent increasing N availability. However, how leaf N per unit mass changes with light availability is not straightforward, with among‐species patterns suggesting higher N in ‘fast’ sun‐adapted species than in ‘slow’ shade‐adapted species—a pattern that would lead to increasing leaf N with increased fire frequency, opposite from the prediction based on soil N alone. Yet within‐species adjustments of leaf N may counteract the broad among‐species pattern (Anderegg et al., 2018). The potential confounding effects of light and soil N availability on SLA, with the potential for intraspecific changes to be in the opposite direction as interspecific changes (e.g. Lusk et al., 2008), make SLA even harder to predict a priori. The values of both of these traits impact multiple ecosystem processes like productivity and decomposition (Cornwell et al., 2008; Hobbie, 2015), making the resolution of these trends relevant to the response of the entire ecosystem.

We tested how repeated burning changes the resource use strategies of plants by sampling foliar SLA and N content along a savanna forest ecotone in a fire‐frequency manipulation experiment ongoing for 41 years at the time of sampling at the Cedar Creek Ecosystem Science Reserve in central Minnesota, USA. We measured SLA and foliar N content in 89 plant species across 905 individuals spanning 12 plots that had received 9 levels of fire frequency treatments. Soil N and light availability were measured in parallel with the trait sampling as was species cover, allowing us to calculate community‐weighted means of each trait in each plot. We addressed four questions:

1. Does fire create a gradient in the availability of light and N and is this linked with a shift in plant community composition and diversity?
2. Is there a functional change in the plant communities that reflects the changes in light and N and how consistent is this change across species to community scales?
3. Are the changes in traits influenced and/or constrained by either phylogeny and/or covariation between leaf N and SLA?
4. Do changes in functional traits track changes in plant biodiversity?

## METHODS

### Study site

The Cedar Creek Savanna Fire Experiment (CCSFE) was established in 1964 in an oak savanna landscape with 17–39% tree cover (latitude: 45.4013°, longitude: −93.1995°). CCSFE experiences a temperate mesic climate (780 mm mean annual precipitation and 6.72°C mean annual temperature, averaged from 1987 to 2016 at the Cedar Creek weather station). Prior to the establishment of the reserve in 1942, fires in the grassland and savanna occurred roughly every 17 years, on average, until c. 1920 (Leys et al., 2019), after which fire was excluded for c. 40 years until the CCSFE was established. In 1964, plots 2.4–18.4 ha large were distributed across a c. 300 ha landscape, separated by fire breaks and assigned fire treatments. We refer to these as units. We focused on 12 units that span the entire fire frequency gradient: unburned (*n* = 3), burning c. once per decade (*n* = 2), c. once every 3 years (*n* = 2), c. once every other year (*n* = 2), and c. three times every 4 years (*n* = 3), generating a fire frequency gradient of 0–0.77 fires/year. We present data in terms of realised fire frequencies because the target frequencies were not always achieved due to variability in burning condition suitability.

Fires are low intensity (flame heights of <1 m) prescribed burns conducted in the spring when the ground is still somewhat moist (April–May). The units are on well‐drained and sandy soils (<8% clay) with similar textures and depths.

### Surveys of plant composition and biomass

Starting in 1995, one permanent plot (50 × 75 m) was established in each of the 12 units. Within each permanent plot, 24 permanent sampling points, placed at 10‐m intervals along transects in the plots, were surveyed every 5 years for herbaceous and shrub cover and composition using a 0.5 m^2^ quadrat between June and August (we used the 2005 survey to align with trait, light, and soil N sampling). Cover classes were estimated for each species in the herbaceous layers using a modified Domin scale with no upper limit on total cover. We calculated continuous cover for each species based on the average within each Domin scale. We averaged the cover of species across all 24 permanent sample points; thus, we calculated diversity over 12 m^2^ and trait means using cover averaged across all 24 points.

### Leaf trait sampling

In 2004–2005, we measured foliar N and SLA on 905 individuals spanning 89 plant species. Species were distributed across the major plant groups in the experiment: N‐fixing forbs (*n* = 4), C3 and C4 grasses (*n* = 20), non‐fixing forbs (*n* = 44), shrubs (*n* = 14), trees (*n* = 5) and vines (*n* = 2). The asymmetry in the number of species among groups arises because species richness differed among groups. We sampled any species with greater than or equal to 0.5% cover in the point in question and which had a healthy leaf available. Composite samples from the same individual were taken to ensure enough material for %N analyses and to have a better estimate of the average leaf than picking a single leaf. The average number of leaves sampled was ca. 4. (see Supplemental Information, SI, for a discussion of sampling relative to the timing of past fires). Individual species' traits were thus measured in all plots if a given species was present. Samples were oven dried, ground through a 40 mm screen and analysed for per cent C and N via combustion on an elemental analyser (ECS 4010, COSTECH Analytical Technologies Inc.). The herbaceous species included in the trait sampling accounted for >80% of herbaceous biomass in the plots.

### Light availability and soil nitrogen

In 2005, light availability was assessed at each of the 24 permanent sample points using a pair of LICOR LAI‐2000 sensors (LICOR Inc., Lincoln, NE). Eight sensor readings under diffuse light were taken at each sample point using a 270° lens (90 occluded) below the tree, shrub, and herbaceous strata and compared with readings from a sensor in a nearby open field. We calculated the difference in interception beneath the different strata to quantify light availability to and interception within the herbaceous layer and averaged all values across points within a plot.

Soil net N mineralisation rates, from 0 to 15 cm in the mineral horizon, were estimated on the permanent plots from 2000 to 2004, with each year containing five incubation periods approximately 30–45 days each throughout the active growing season (mid‐April to mid‐October) at eight replicate points nearby the herbaceous surveys; we summed the values across all five incubation periods to get a total N mineralisation for the active growing season. Detailed methods for assays and laboratory analyses are described in Reich et al. (2001) and the Supporting Information. At each point, a 15‐cm long soil core was removed from the sample area and served as the initial sample. We used the measurements of inorganic N on the initial sample to calculate total standing inorganic N. We calculated stocks and fluxes using bulk density on oven‐dried soil taken in each plot taken in 2002.

### Phylogenetic tree

A phylogenetic tree was constructed using the GBOTB_extended.tree (Jin & Qian, 2019) as a backbone, an updated version of the Spermatophyta mega‐phylogeny GBOTB (Smith & Brown, 2018). Missing species were added to their respective genera and families using the R package *V.PhyloMaker* (Jin & Qian, 2019). Branch lengths of the imputed species were set using the BLADJ approach by placing terminal branches at the midpoint of their sister lineages.

### Statistical analyses

#### Fire effects on light and nitrogen

The change in light availability across the fire frequency gradient was analysed using a polynomial regression to capture the non‐linear relationship between fire frequency and light availability. We used a mixed‐effects model only for inorganic N and N mineralisation because they were sampled annually for 5 years to account for inter‐annual variation, using plot as the random intercept and fire frequency as a fixed effect. All analyses were performed in R (R Development Core Team, 2010) and the mixed‐effects models for inorganic N and net N mineralisation used the package *lme4* (Bates et al., 2015).

#### Community composition and diversity

The composition and cover of herbaceous species were evaluated by averaging across all points within a plot for consistency with the scale at which we calculated the community‐weighted mean trait values (see below). Marginal effects of fire, light availability and net N mineralisation on community composition were analysed using a PERMANOVA on Bray–Curtis dissimilarity, using marginal (Type III) sums of squared calculations in the R package *vegan* (Oksanen et al., 2007). Species richness, Shannon–Wiener diversity, Pielou species evenness and phylogenetic diversity (sum of branch lengths, (Faith, 1992)) were analysed as a function of fire frequency with a quadratic regression.

#### Trait community‐weighted means

We calculated the community‐weighted mean (CWM) for traits at four hierarchical scales to partition the roles of functional group, genus, species and intraspecific variability in driving the turnover in traits across the fire gradient. We used linear models to analyse the relationship between fire frequency and traits because a single CWM was calculated for each plot using the averaged cover and composition across all points within a plot. The hierarchical scales included: (i) full variation: using traits measured at each plot for each species present weighted by the relative cover of each species, in order to capture all sources of variation including within‐species variation across the fire gradient (ii) species means: using a single average trait value across all plots for each species, weighted by each species' relative cover to exclude the effect of within‐species variation but capture the effects of species turnover and changes in abundance (iii) genera means: aggregating species to genera and using a genus‐averaged trait value weighted by genus relative cover, in order to capture only turnover or abundance changes in plant genera (iv) functional type means: aggregating species to functional groups and using a functional group‐averaged trait value weighted by the relative cover of each functional group in each plot, in order to capture only changes in functional group composition.

We tested the degree to which changes in CWMs are attributed to changes in the relative phylogenetic similarities of plant communities in plots using Blomberg's K with a comparison to a Brownian Motion evolutionary model (see Supporting Information for detailed methods) (Blomberg et al., 2003). An observed K value that falls within the distribution of a Brownian Motion model of evolution indicates that the model explains the trait variation among species well and indicates a degree of phylogenetic conservatism. K values that fall within the distribution of a white noise model indicate that evolutionary history does not explain trait variation and that traits are labile.

#### Trait covariation

To evaluate the covariation between traits, we fit ordinary least squares regressions and tested their significance using Type II linear models between foliar %N and SLA using package *lmodel2*. To test how fire treatment changed the coupling between traits and whether that differed across functional groups, we fit models within each fire treatment, and then within each functional group in that fire treatment. Correlations between traits were also analysed using phylogenetic regressions (Freckleton et al., 2015). Lambda (λ) represents the degree of phylogenetic dependence of the data varying between 0 (phylogenetic independence) and 1 (species' traits covary in direct proportion to their shared evolutionary history).

## RESULTS

### Frequent burning increases light and decreases soil N availability

**FIGURE 1:**
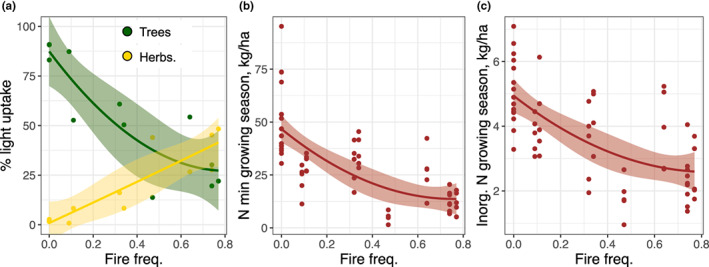
Frequent burning created a gradient in light and nitrogen (N) availability. (a) Light uptake measured as the percentage of the decline in photosynthetically absorbed radiation beneath the different strata of vegetation (e.g. ‘trees’ indicates the penetration through the tree canopy). (b) Net N mineralisation and (c) standing inorganic N during the growing season (May–October) replicated across 5 years. Standing inorganic N calculated by averaging the initial N measurements conducted during the mineralisation assays. Lines are polynomial regressions with shading displaying the standard error of the fit. Fire frequency denotes the number of fires per‐year.

Fire frequency created a strong gradient in light and N availability. Fire exclusion resulted in the formation of forest with ≈12% light penetration to the herbaceous layer while nearly annual burning maintained a savanna with ≈76% light penetration (*F*_1,10_ = 29.2, *p* < 0.001, linear regression; Figure 1a, Table S1). Greater light penetration below tree canopies results in denser herbaceous cover (Figure 1a) and more understory biomass (Pellegrini et al., 2019; Reich et al., 2001). Fire reduced both the cycling and stocks of soil inorganic and total N. Net N mineralisation and inorganic N in the growing season declined quadratically from 5.15 to 1.15 kg N ha^−1^ and from 0.51 to 0.24 kg N ha^−1^, respectively, from fire exclusion to the highest frequency (*F*_2,53_ = 31, *p* < 0.001 and *F*_2,53_ = 28.8, *p* < 0.001, respectively, mixed‐effects models; Figure 1b,C Table S1). Average stocks of total soil N also declined from 2.75 to 1.68 Mg N ha^−1^ across the fire gradient (*F*_1,10_ = 6.0, *p* = 0.034, linear regression; Table S1). Consequently, frequent burning maintained a high‐light but low‐N environment.

### Fire frequency and shifts in light and nitrogen change species composition

**FIGURE 2:**
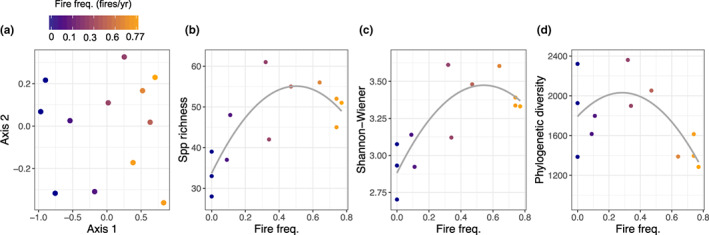
Changes in composition and species diversity across the fire frequency treatments (fires/year). (a) nonmetric multi‐dimensional scaling on a Bray–Curtis community dissimilarity matrix, where each point is a plot coloured by fire frequency treatment (stress = 0.066). (b–d) Diversity metrics as a function of fire frequency (units in number of fires per‐year) based on averages over 12 m^2^. Regression statistics on 2nd order polynomial: species richness, *r* ^2^ = 0.62, *F*_2,9_ = 9.9, *p* = 0.005; Shannon–Wiener species diversity, *r* ^2^ = 0.64, *F*_2,9_ = 10.8, *p* = 0.004; phylogenetic diversity, *r* ^2^ = 0.36, *F*_2,9_ = 4.1, *p* = 0.055.

Fire frequency significantly changed the diversity and composition of the herbaceous community. First, fire frequency treatments alone explained 30% of the variance in community composition dissimilarities across plots (ordination, *F*_1,11_ = 4.23, *p* = 0.001, Figure 2a). These changes occurred via increases in the cover of grasses, non‐leguminous forbs and leguminous forbs from increasing fire frequency, with shrubs and small trees in the herbaceous layer remaining unchanged (Figure S1). Light and in situ net N mineralisation together explained 23% of the variance, with light explaining more variance than N, when considered individually (*r* ^2^ = 0.14 vs. *r* ^2^ = 0.09, respectively).

Species richness and diversity exhibited quadratic relationships with fire frequency, such that they were lowest in the fire exclusion plots, peaked at an intermediate (fires every 3 years) frequent burning, but began to decline at the highest frequencies (Figure 2b,c). The declines in the most frequently burned plots were small enough that they maintained higher species diversity (+22%) and richness (+43%) than fire exclusion plots. Evenness displayed no significant relationship with fire (*r* ^2^ = 0.07, *F*_2,9_ = 1.4, *p* = 0.30). Phylogenetic diversity peaked at slightly lower fire frequencies, declining sharply at high fire frequencies such that the most frequently burned plots had 24% lower phylogenetic diversity than fire exclusion plots and 33% lower diversity than plots burned once every 3 years (Figure 2d). When soil N and light availability were directly compared with diversity and richness, we found all metrics declined with increasing soil net N mineralisation and decreasing light availability (all *p* < 0.05, Figure S2) except phylogenetic diversity (N mineralisation: *p* = 0.45; light: *p* = 0.46).

Thus, greater soil N and lower light arising from fire exclusion were related with both unique and less species‐diverse plant communities.

### Frequent fire selects for conservative resource use strategies

**FIGURE 3:**
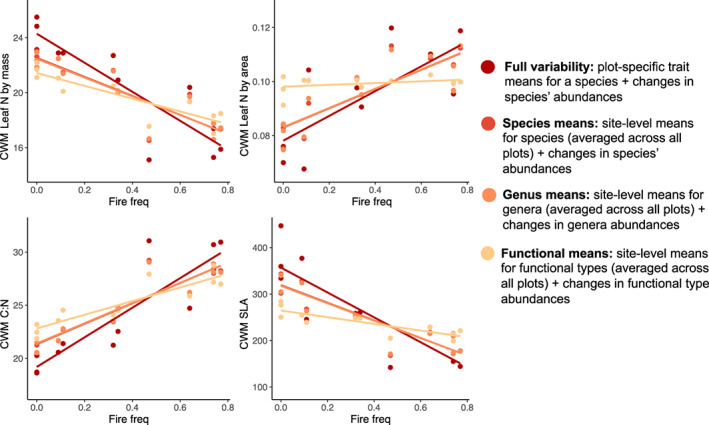
Changes in leaf nitrogen by mass (N, mgN/g‐dry mass) and area (mgN/cm^2^‐leaf area), carbon to nitrogen ratio (C:N), and specific leaf area (SLA, cm^2^/g) across the fire frequency gradient (fires/year). Community‐weighted mean (CWM) traits for each plot were calculated using trait values aggregated to four different levels: using trait values measured for each species in each plot (‘Full variability’), using species average trait value across all plots (‘Species' means’, which excludes within‐species variation), using a single genus‐average trait value for all species in a genus (‘Genera means’) and using a single average trait value for all species in a functional group (‘Functional means’) (so that trait change was only driven by composition turnover) and incorporating intraspecific variability. Shading represents standard errors in the linear regression fit.

More frequent burning resulted in plant communities having lower CWM leaf N concentrations by mass, higher C:N ratios, lower SLA and higher leaf N by area than less frequent burned plots. These changes emerged due to both intraspecific phenotypic variability as well as turnover in the composition of species, genera and functional groups with different trait means (Figure 3, Table S2).

When CWMs were calculated with the full variability in trait values (including intraspecific variation in traits among plots, dark lines in Figure 3), CWM leaf N declined linearly from 24.5 mg N/g leaf in unburned forests to 21.8 mg N/g leaf in intermediately burned savannas, and 16.2 mg N/g leaf in the most frequently burned savannas (*p* < 0.001, Table S2). Leaves became thicker and/or denser, with CWM SLA declining from 380 cm^2^/g in unburned forests to 259 cm^2^/g in intermediately burned savannas to 172 cm^2^/g in the most frequently burned savannas (*p* < 0.001, Figure 3, Table S2). Thus, fire selects for more conservative resource use strategies at multiple scales.

### Reinforcing changes within species and across species, genera and functional types

We found changes in functional traits were reinforced at all hierarchical scales, creating a steeper trait–fire relationship as we incorporated finer‐scale variability in traits across the gradient (*p* < 0.001 for all traits; Figure 3, S3, Table S2). For example, when we accounted for intraspecific changes and turnover in species composition, CWM foliar %N and SLA were 51% and 122% higher in the fire exclusion relative to the most frequently burned plots. In contrast, using functional form trait means and re‐calculating the CWM based only on changes in their relative abundance created a gradient where foliar %N and SLA were only 21% and 28% higher in the fire exclusion relative to the most frequently burned plots. We refer to the phenomenon of reinforcing changes at the different hierarchical scales as a ‘co‐gradient’.

**FIGURE 4:**
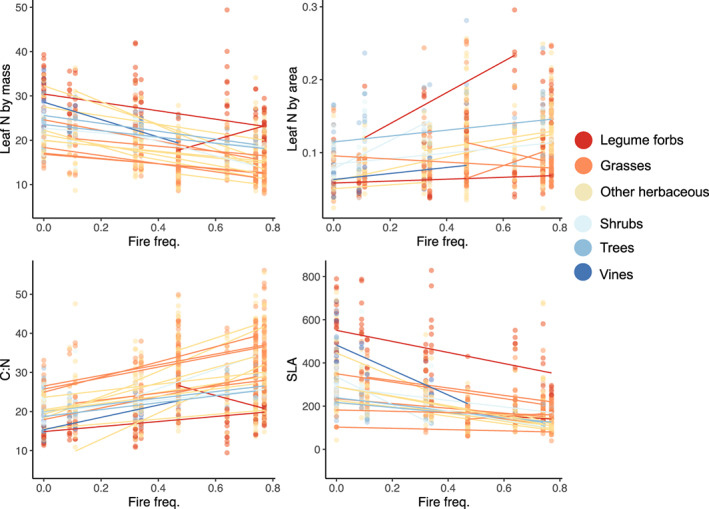
Species‐level changes in leaf N by mass (N, mgN/g‐dry mass) and area (mgN/cm^2^‐leaf area), carbon to N ratio (C:N) and specific leaf area (SLA, cm^2^/g) across the fire frequency gradient (fires/year) (*n* = 89 species total with *n* = 905 replicate individuals). Points and lines are coloured by their functional group with lines indicating regression fits within species at *p* < 0.10.

One key driver of the co‐gradient was the role of intraspecific variability in species that occurred in multiple fire treatments across the fire gradient. For example, the slope coefficients for foliar %N and SLA were 52% and 41% greater, respectively, when intraspecific changes were incorporated relative to interspecific changes alone (Table S2). Additionally, trait–fire trends within species (not considering changes in % cover of the species) across fire treatments, illustrated that fire frequency correlated with intraspecific changes in foliar N in 22 species and SLA in 26 species, with 21 and 24 species exhibiting declines with more frequent burning, respectively (*n* = 89 total, Figure 4); but many of the remaining species were rare. These consistent changes from the species to community levelssupport our hypothesis that fire‐driven changes in N and light are reflected by species' strategies at the individual to community scales.

### Attributing variation in traits and their turnover

Although the trait–fire slope became increasingly steeper as we incorporated finer‐scale changes in traits, the magnitude of the increase was most pronounced when moving from functional type‐level averages to genus‐level averages and then from species‐level averages to within‐species variability (Figure 3). Moving from genus‐level averages to species‐level averages barely changed the trait–fire slope, illustrating that turnover among congeners was less important than turnover in genera and within‐species trait variation.

The turnover in plant community phylogenies was an important factor driving trait changes. Fire‐driven changes in phylogenetic groups explained 52% and 49% of the variance in SLA and foliar N by mass across the fire gradient (Bayesian phylogenetic regressions, Figure S5, Table S4). Consequently, both ecological and evolutionary factors contributed to the observed changes in functional traits of the plant community.

Analyses of phylogenetic signals within species illustrated some evidence of phylogenetic conservatism for all foliar traits (Table S3). When comparing Blomberg's K with the white noise model, evolutionary history explained trait variation. Comparisons with the Brownian motion model also demonstrated significantly different K values, illustrating a degree of phylogenetic conservatism.

### Covariation among traits depends on fire, functional groups, and evolutionary history

**FIGURE 5:**
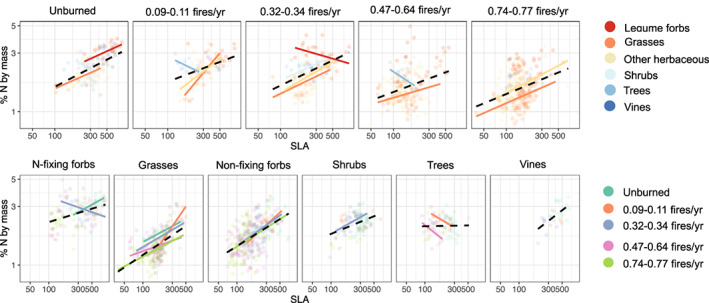
Correlations between specific leaf area (SLA, cm^2^/g) and foliar nitrogen by mass (%N) calculated at two categorical scales: (a) by functional group (across fire frequencies) and (b) by fire frequency (across functional groups). The black dashed line is fit to all data within the panel while the coloured lines are fit within the categories in the panel (e.g. grasses in unburned plots). Only significant relationships are displayed.

Covariation between foliar N and SLA was significant within each fire treatment (rho spanned 0.32–0.53, *p* < 0.001) and within some individual functional groups within each fire treatment (Figure 5), especially within grasses and non‐N‐fixing forbs (Figure 5, Table S5). Across all species, we found varying degrees in how much species' traits covariance was due to their phylogenetic relatedness. The phylogenetic dependence of the relationship between SLA and N had a lambda value of 0.75 (*r* ^2^ = 0.51, *p* < 0.001), illustrating a greater influence of phylogenetic similarity than independence because a lamba = 1 indicates a complete covariation in proportion to phylogenetic similarity (Table S5). Across all trait combinations, covariance remained significant when we accounted for phylogenetic similarity.

### Weak relationship between changes in species diversity and functional traits

**FIGURE 6:**
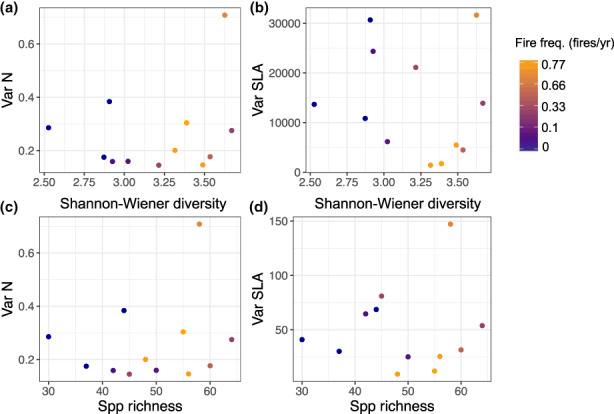
Variation in traits within communities are decoupled from the plant community diversity. Scatter plots of the variation in leaf nitrogen by mass (N, %) and specific leaf area (SLA, cm^2^/g) as a function of species diversity and fire frequency (fires per‐year). Points are coloured by fire treatment. Variance is calculated as the square of the standard deviation.

We found weak evidence that variation in foliar traits among individuals in a community correlated with species or phylogenetic diversity in plots or fire frequency. The lack of correlation illustrates a decoupling between functional and compositional diversity (Figure 6; Figure S6 has probability distributions of the traits). Consequently, more frequently burned and species‐diverse plots did not intrinsically have a greater diversity of functional traits.

## DISCUSSION

Fire frequency changed the floristic and functional composition and diversity of plants, which was  correlated with fire‐driven shifts in light and soil N. There were strong associations between more frequent burning and  declines in SLA and foliar N concentrations both within species and arising through the turnover in the composition of species, genera and functional groups, pointing to fire‐driven changes in light and soil N placing a strong selective pressure on plant species and communities. Although certain phylogenetic groups may occupy specific trait–fire niches, the large intraspecific changes and turnover in functional groups allowed community‐level trait changes to be relatively unconstrained by the present phylogenetic groups.

### Eco‐evolutionary causes of fire‐driven differences in community traits

Both ecological and evolutionary variables regulated the change in the traits of the plant community. Ecologically, the strong decreases in soil N and increases in light availability with more frequent burning influenced the turnover in the composition of plants with different traits and intraspecific variability in traits. First, we observed declining CWM foliar N concentrations with increasing fire frequency (Figure 3). Second, frequent burning resulted in herbaceous communities dominated by species with conservative nitrogen‐use strategies, such as C4 grasses with higher C:N ratios (Figures S1, S4) (Tjoelker et al., 2005). Third, traits of species that occurred in multiple plots changed in the same direction as the CWMs, such that foliar N concentrations and SLA within species declined with more frequent burning (Figures 3 and 4). Taken together, the strong selection for conservative resource use strategies emerged consistently at the community and species scales, leading to increasingly steep trait–fire relationships when multiple scales were considered (Figure 3).

Evolutionary constraints also influenced the CWM trait changes. First, incorporating phylogenetic information increased the variance in traits explained by fire (Table S3). Second, Blomberg's K values for foliar N and SLA significantly differed from the white noise model, indicating that evolutionary history explains some trait variation. However, there was a low degree of phylogenetic conservatism because K values for traits were significantly different than what would be expected under a Brownian motion evolutionary model. Few of the previous studies on trait turnover along savanna forest ecotones have explicitly considered the role of phylogeny (Hoffmann et al., 2012; Lehmann et al., 2011; Pellegrini et al., 2015; Reich et al., 2001; Schmidt et al., 2011), despite the plant lineages in savanna forest ecotones differing among continents (but see (Cavender‐Bares & Reich, 2012)). A better understanding of the composition of plant clades may provide insight into the inconsistent functional changes along ecotones, which is unexplained by existing studies.

### Implications for understanding trait–environment relationships more broadly

The existence of similar correlations between traits and fire frequency across multiple hierarchical scales was not expected a priori, given that selection for trait combinations at the community scale often differ from the scale at the individual species (Anderegg et al., 2018; Cornwell & Ackerly, 2009; Dwyer et al., 2014). One hypothesis for decoupling between intra‐ and interspecific changes is that non‐monotonic trait–environment relationships within species lead to positive or negative trait–environment correlations at the extremes of species' distributions that contrast with correlations among species (Albert et al., 2010). In our case, environmental conditions may be relatively extreme such that all within‐species trait patterns are monotonic with increasing fire frequency but would not be so under less limiting nitrogen conditions. Another hypothesis is that plasticity (contributing to within‐species variation) and adaptive evolution (underpinning among‐species variation) allow trait–environment and even trait–trait relationships within species to become decoupled from among‐species patterns (Ackerly & Cornwell, 2007; Anderegg et al., 2018; Messier et al., 2017). The strong and consistent intraspecific trait changes in leaf N and SLA found here (Figure 4) that reinforce genus and species replacement across the fire gradient are somewhat rare in the literature (Cornwell & Ackerly, 2009; Rosas et al., 2019; Schulze et al., 1998) suggesting a remarkably consistent selection for lower leaf N and SLA with increasing fire across multiple modes of trait change (plasticity, species turnover, lineage turnover).

We also found fire shifted CWMs at taxonomic scales consistent with those observed at intraspecific and community scales (Figure 3). For example, more frequent fires consistently selected for more resource conservative clades and conservative species within those clades and conservative individuals within those species. The consistency of trait variation across taxonomic and ecological scales is relatively rare in observational studies utilising environmental gradients (Albert et al., 2010; Anderegg et al., 2018; Cornwell & Ackerly, 2009; Dwyer et al., 2014). We did find that covariation between traits tended to be weaker within plots (Figure 5), potentially due to a lower degree of environmental differences that could select or filter for species' strategies than across the fire gradient. Thus, repeated disturbance is a likely driver of community assembly and evolutionary selection with  leaf traits being important for species' adaptations to changes in light and soil N.

We focused on a relatively limited number of traits—in contrast, trait syndromes are defined by a larger suite of traits (Reich, 2014; Reich et al., 1997; Wright et al., 2004). For example, belowground traits such as root morphology, chemistry and microbial symbioses create a spectrum along a conservative–acquisitive axis and another along an axis of reliance on microbial symbionts (Bergmann et al., 2020). We would expect that belowground traits may also respond to the fire‐driven changes in soil nutrients to a similar degree as leaf traits, but the syndromes related to microbial symbionts may be more phylogenetically conserved (Bergmann et al., 2020) than what we observed for foliar traits. The lack of change in leaf traits within some species could be because they vary in belowground traits that allow them to maintain the same leaf traits despite shifts in resources.

### Decoupling between compositional and functional diversity

Given the strong relationship between fire‐driven changes in light and soil N and functional turnover, we expected that both resources would shape species' niches along the fire gradients. While we did find light and soil N changed the relative abundance of species with different functional traits, we did not find that functional trait variability correlated with floristic diversity. The lack of correlation may be because (i) trait changes were driven by intraspecific phenotypic variability and turnover in plant genera, not necessarily species, and (ii) multiple species had similar trait values, but shifted in abundance across the gradient, creating functional redundancy. One caveat is that biodiversity and trait variation could be driven by unmeasured traits.

### Implications for fire‐nitrogen feedbacks

Fire‐driven N losses are hypothesised to generate positive feedbacks: frequent burning volatilises N, reducing plant growth and filtering for conservative strategies, which further reduces N turnover and ability of certain taxa, especially trees, to re‐grow after fire and form a forest (Reich et al., 2001). Our results suggest this feedback is likely occurring and is being driven by both species turnover and the phenotypic responses of individual species. The decline in photosynthetic capacities and growth rates associated with species having lower SLA and foliar N (Reich, 2014), including among savanna and forest species at this site (Reich et al., 2003; Tjoelker et al., 2005), would lead to slower N cycling from plants into the litter layer. Because litter N content is the primary determinant of litter mass loss in this savanna (Hernández & Hobbie, 2008), these trait changes lead to lower turnover from litter into mineral soils. Thus, if fire filters for species or phenotypes with a higher C:N ratio, such as C4 grasses, then decomposition and turnover of N will decline, and immobilisation of N will rise. Nitrogen turnover may decrease further with declining soil N, because soil N tends to be positively related to litter decay rates (Hernández & Hobbie, 2008) and the activity of extracellular enzyme involved in the decomposition of soil organic matter (Pellegrini et al., 2020). Our findings suggest the potential for fire‐nitrogen feedbacks is structured not only via the response of trees but also changes in the traits of the herbaceous community.

## AUTHOR CONTRIBUTIONS

A.F.A.P., L.A., S.E.H., and P.B.R. conceived of the study with input from J.C‐B. S.E.H. and P.B.R. designed the experiment and sampling methods. A.F.A.P., L.A., J.N.P‐L., and J.C‐B. performed statistical analyses. A.F.A.P. wrote the first draft of the manuscript with feedback from all co‐authors.

## Supporting information


Supporting information S1.

## Data Availability

All data and code are available: https://doi.org/10.6084/m9.figshare.21572373
