# Supplementary material for: Consistent physiological, ecological and evolutionary effects of fire regime on conservative leaf economics strategies in plant communities
Source: Ecol Lett. 2023 Feb 23;26(4):597–608. doi: 10.1111/ele.14182 (PMC10947573; doi:10.1111/ele.14182)
Supplement: Supplementary file 1 — Supporting information S1. [file ELE-26-597-s001.docx]

**Consistent physiological, ecological, and evolutionary effects of fire regime on conservative leaf economics strategies in plant communities**

Adam F. A. Pellegrini, Leander Anderegg, Jesús N. Pinto-Ledezma, Jeannine Cavender-Bares, Sarah E. Hobbie, Peter B. Reich

**Supplemental Information**

*Soil nitrogen stocks and mineralization*

Eight replicates were measured in each plot nearby the points used in the herbaceous surveys. At each point, a 15 cm long soil core was removed from the sample area and served as the initial sample. A paired semi-open core was placed within 10 cm of the initial sample by inserting a 15 cm pipe drilled with air holes and with a plugged top. At the end of the incubation period, samples were hand homogenized and passed through a 2 mm sieve. Soil NO_3_^-^, NO_2_^-^, and NH_4_^+^ were extracted in 50 ml of 1M KCl solution and analyzed colorimetrically. We used values from the initial samples to calculate the standing inorganic N concentrations in this study. Mineralization rates were calculated over the entire growing season. We calculated stocks and fluxes using bulk density on oven-dried soil taken in each plot.

*Further information on trait surveys*

A full list of species and their associated sample sizes can be found in Table S6. Time since fire at the time of sampling differed across plots ranging from c. 3 months to >43 years (exclusion plots). While the time since fire negatively correlated with fire frequency treatment (*r^2^*=0.54), this was mostly driven by the plots with decadal burns or unburned. In fact, there was no relationship between time since fire and fire frequency for the seven plots with fires ranging once every three years to three times every four years (*r^2^*=0.02). Given the trait-fire relationship emerged even within these relatively frequently burning plots, we do not expect time since fire to have influenced our results.

All the sampled plants were from the ‘ground layer’ in order to best match the light conditions measured and to fit into the context of asking about traits along light, fire, and N gradients; meaning no leaves sampled from tall mature trees. Shrubs and trees were likely neither seedlings nor juveniles, but resprouts with root systems likely five to many years old, and stems just a few years old.

*Phylogenetic analyses*

The overall degree to which trait values of species depended on phylogenetic relatedness was tested using Blomberg’s K (using a white noise null model on 1,000 randomized samples) with a comparison to a Brownian Motion evolutionary model (1,000 simulated traits) (Blomberg *et al.* 2003) as well as to a white noise null model in species trait values are independent of their phylogenetic relationships. An observed K value that falls within the distribution of a Brownian Motion model of evolution indicates that the model explains the trait variation among species well and indicates a fairly high degree of phylogenetic conservatism. K values that fall within the distribution of a white noise model indicate that evolutionary history does not explain trait variation, and that traits are labile.

Table S1: Environmental gradient in light and soil nitrogen resulting from repeated burning. Light=% penetration measured via a LICOR LAI 2000. N min (nitrogen mineralization) =kgN/ha across the growing season, mid-April to mid-October. Inorganic N = kgN/ha. Carbon and nitrogen = Mg/ha down to 20 cm.

| **Fire freq.** | **Light** | **sd** | **Nmin** | **sd** | **Inorg N** | **sd** | **Carbon** | **sd** | **Nitrogen** | **sd** |
| --- | --- | --- | --- | --- | --- | --- | --- | --- | --- | --- |
| 0 | 11.8 | 4.5 | 5.15 | 0.76 | 0.51 | 0.02 | 44.56 | 12.50 | 2.75 | 0.76 |
| 0.09-0.11 | 30.0 | 24.4 | 2.68 | 0.60 | 0.40 | 0.04 | 39.93 | 4.17 | 2.33 | 0.16 |
| 0.32-0.34 | 44.4 | 7.4 | 3.29 | 0.43 | 0.39 | 0.08 | 43.72 | 6.44 | 2.72 | 0.17 |
| 0.47-0.64 | 66.0 | 28.7 | 1.57 | 1.51 | 0.29 | 0.15 | 32.29 | 11.56 | 2.00 | 0.73 |
| 0.74-0.77 | 76.1 | 5.6 | 1.15 | 0.30 | 0.24 | 0.02 | 27.64 | 3.10 | 1.68 | 0.11 |

Table S2: Community-weighted means for leaf traits in two statistical models either using full trait variability or holding within-species’ means constant. df=1,10 and p<0.01 for all comparisons except for a p=0.036 for fixed species’ means N by area.

| Full variability | | |  |  |  | Species’ means | | |  |
| --- | --- | --- | --- | --- | --- | --- | --- | --- | --- |
| **Trait** | **Intercept** | **Coefficient** | **r^2^** | **F** |  | **Intercept** | **Coefficient** | **r^2^** | **F** |
| N by mass | 2.43 | -1.05 | 0.75 | 34.28 |  | 2.25 | -0.69 | 0.76 | 35.25 |
| N by area | 0.08 | 0.05 | 0.56 | 14.7 |  | 0.08 | 0.04 | 0.68 | 43.60 |
| CN | 19.20 | 13.92 | 0.77 | 38.80 |  | 21.34 | 9.59 | 0.84 | 58.75 |
| SLA | 357.03 | -267.84 | 0.69 | 26.05 |  | 319.20 | -189.56 | 0.80 | 46.11 |

Table S3: Phylogenetic signal using Blomberg’s K. K p value represents p values against a randomization test in which the phylogeny tips (with 1,000 randomized samples; white noise null model) are shuffled. BM p value, the p values represent the probability of the observed K value being greater or smaller (1000 simulated traits) than a Brownian motion (BM) evolutionary model. A K p value <0.05 can be interpreted as (1) evidence for phylogenetic signal and a BM p value >0.05 can be interpreted as (2) consistency with the BM evolution model.

| Trait | K | K p value | BM p value |
| --- | --- | --- | --- |
| %N | 0.16 | <0.01 | <0.01 |
| N_Area_ | 0.25 | <0.01 | <0.01 |
| CN | 0.15 | <0.01 | <0.01 |
| SLA | 0.21 | <0.01 | <0.01 |

Table S4: Bayesian r^2^ from the phylogenetic regressions between fire frequency and the community-weighted trait means.

| Trait | Type | r^2^ |
| --- | --- | --- |
| %N | Marginal | 0.16 |
| %N | Conditional | 0.65 |
| CN | Marginal | 0.20 |
| CN | Conditional | 0.58 |
| N_Area_ | Marginal | 0.03 |
| N_Area_ | Conditional | 0.67 |
| SLA | Marginal | 0.17 |
| SLA | Conditional | 0.69 |

Table S5: Correlations between foliar N by mass and SLA (log_10_ transformed) with the intercept and slope as well as the goodness of fit and test of significance. Ordinary least squares on a Type II ANOVA.

| **Functional group** | **Intercept** | **log_10_(SLA)** | **n** | **rho** | **p value** | **r^2^** |
| --- | --- | --- | --- | --- | --- | --- |
| ForbFixer | 0.04 | 0.16 | 120 | 0.41 | 0.01 | 0.17 |
| Grass | -0.59 | 0.34 | 244 | 0.55 | 0.01 | 0.30 |
| Herb | -0.48 | 0.32 | 306 | 0.52 | 0.01 | 0.27 |
| Shrub | -0.16 | 0.21 | 104 | 0.42 | 0.01 | 0.18 |
| Tree | 0.30 | 0.01 | 78 | 0.02 | 0.43 | 0.00 |
| Vine | -0.60 | 0.39 | 27 | 0.60 | 0.01 | 0.36 |
|  |  |  |  |  |  |  |
| **Fire freq.** | **Intercept** | **log_10_(SLA)** | **n** | **rho** | **p value** | **r^2^** |
| 0 | -0.43 | 0.32 | 160 | 0.53 | 0.01 | 0.28 |
| 0.09-0.11 | -0.22 | 0.23 | 99 | 0.40 | 0.01 | 0.16 |
| 0.32-0.34 | -0.37 | 0.29 | 143 | 0.43 | 0.01 | 0.19 |
| 0.47-0.64 | -0.30 | 0.23 | 193 | 0.33 | 0.01 | 0.11 |
| 0.74-0.77 | -0.36 | 0.25 | 284 | 0.32 | 0.01 | 0.10 |

**Table S6 (***attached***):** List of species surveyed for traits and their associated sample sizes summed across all plots.

Figure S1:


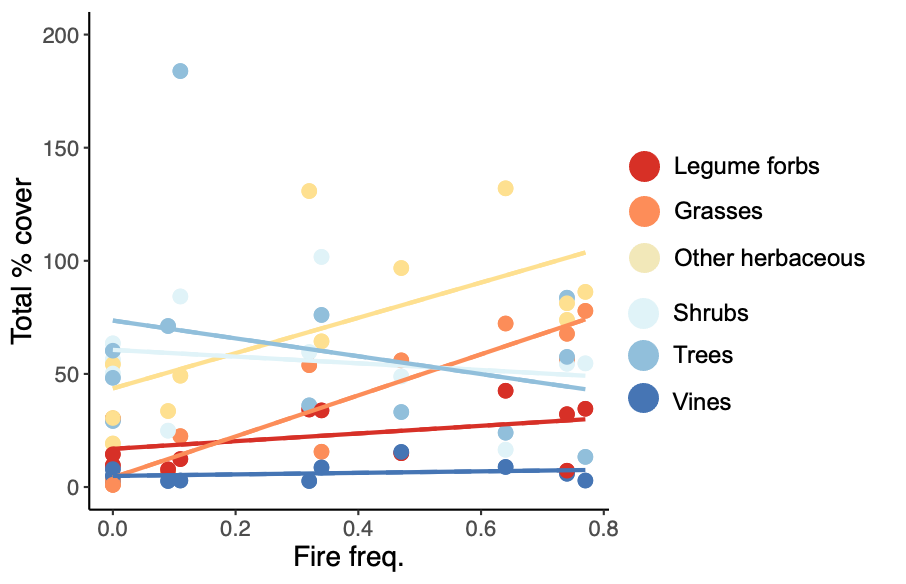


Figure S1: Effects of fire frequency (fires/year) on the relative cover of different functional groups in 2005. Lines are linear regressions within each functional type. ANOVAs on total cover ~ fire frequency; legume forbs, F_1,10_=2.1, p=0.18; grasses, F_1,10_=64.6, p<0.001; herbs, F_1,10_=7.9, p=0.019; shrubs, F_1,9_=0.3, p>0.5; trees, F_1,10_=0.8, p=0.39; vines, F_1,9_=0.62, p=0.45.

Figure S2:

Figure S2: Relationships between plot species richness, Shannon-Wiener species diversity, and Pielou evenness and soil nitrogen (N) mineralization (kgN ha^-1^ across the growing season) and % light penetration. Composition calculations made over a total area of 12 m^2^ in each plot. Lines are linear model fits. Light penetration measured by 1- fraction of photosynthetically absorbed radiation beneath the tree canopy. Net-N mineralization during the growing season (mid-April to mid-October). Light regressions: (species richness, F_1,10_=9.6, p=0.011, Shannon-Wiener species diversity, F_1,10_=5.7, p=0.038; Pielou species evenness, p>0.5). N mineralization regressions: (richness, F1,10=6.0, p=0.034; Shannon-Wiener species diversity, F_1,10_=8.6, p=0.013; Pielou species evenness, F_1,10_=2.7, p=0.13).

Figure S3:


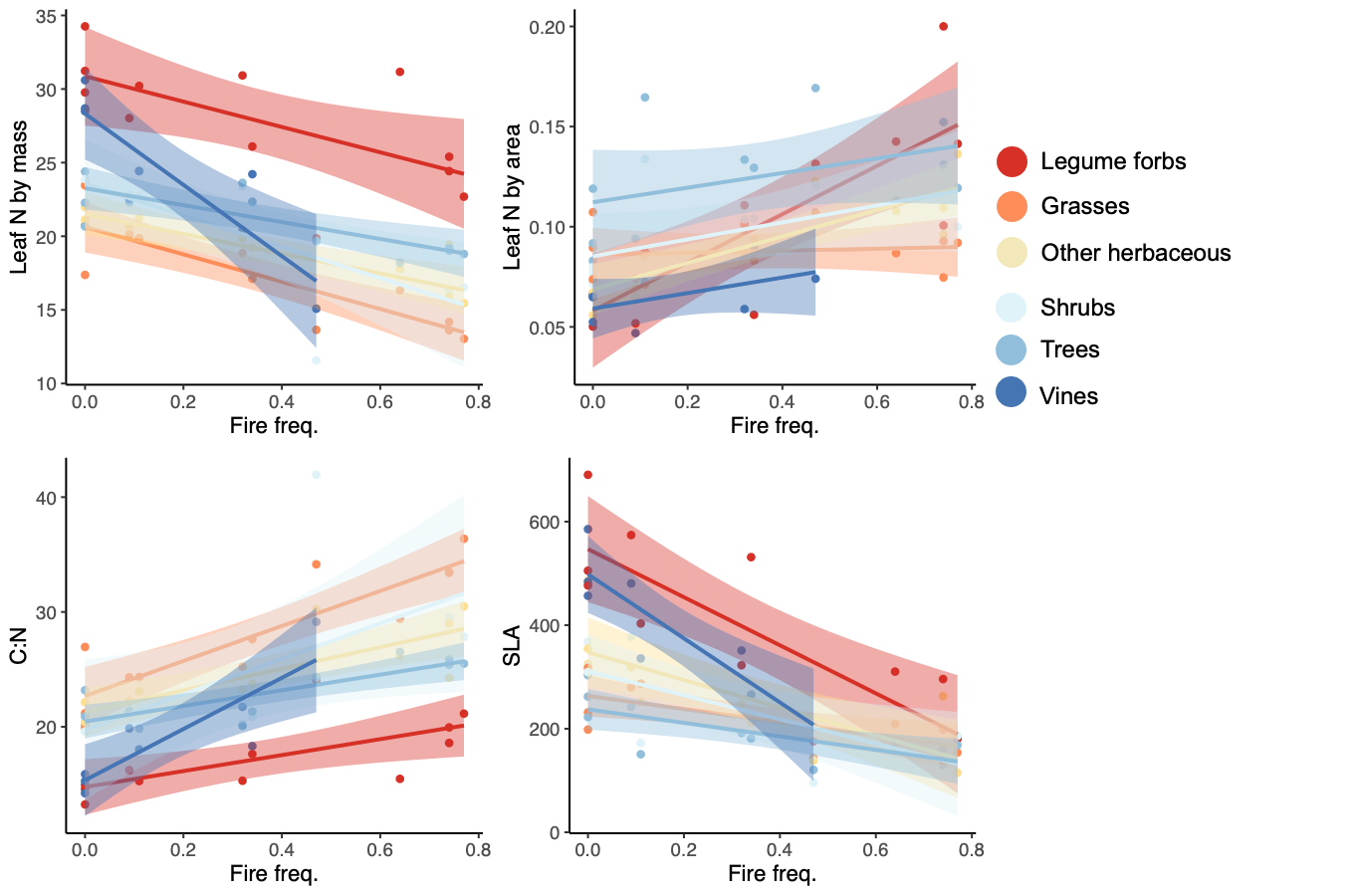


Figure S3: Within-functional group community-weighted mean (CWM) traits across the fire frequency gradient (fires/year) for leaf nitrogen by mass (N, mgN/g-dry mass) and area (mgN/cm^2^-leaf area), carbon to nitrogen ratio (C:N), and specific leaf area (SLA, cm^2^/g). Shading represents standard errors in the linear regression fit.

Figure S4:

Figure S4: Box and whiskers plot of traits within different functional groups (calculated across species-level averages across all fire plots). Leaf nitrogen (N) by mass is %N, specific leaf area (SLA) is cm^2^/g, and leaf N by area is gN/cm^2^-leaf area, C:N is carbon/nitrogen by mass.

Figure S5:


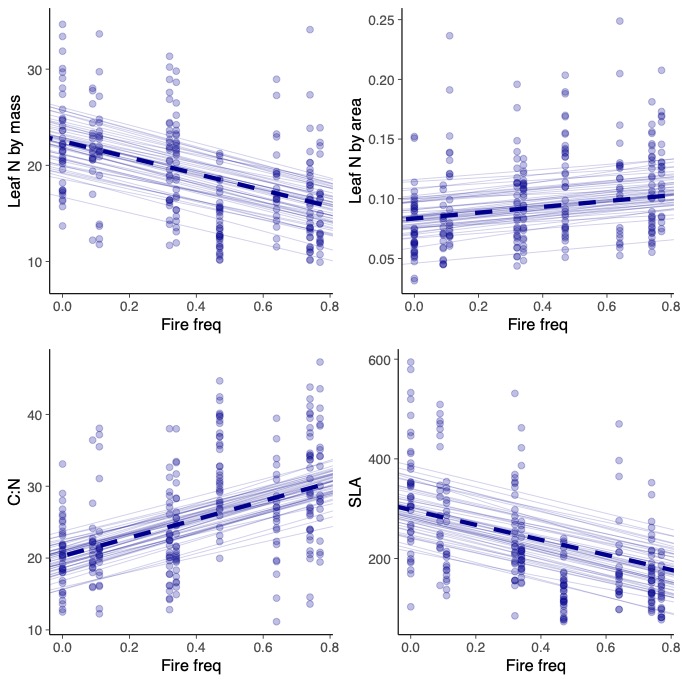


Figure S5: Regressions between species’ mean traits and fire frequency using Bayesian phylogenetic regressions. Changes in leaf nitrogen by mass (N, mgN/g) and area (mgN/cm^2^-leaf area), carbon to nitrogen ratio (C:N), and specific leaf area (SLA, cm^2^/g) across the fire frequency gradient (fires/year). Dashed-tick lines represent the median slope estimation and the continuous-lines 50 slope estimations picked at random from posterior distribution.

Figure S6


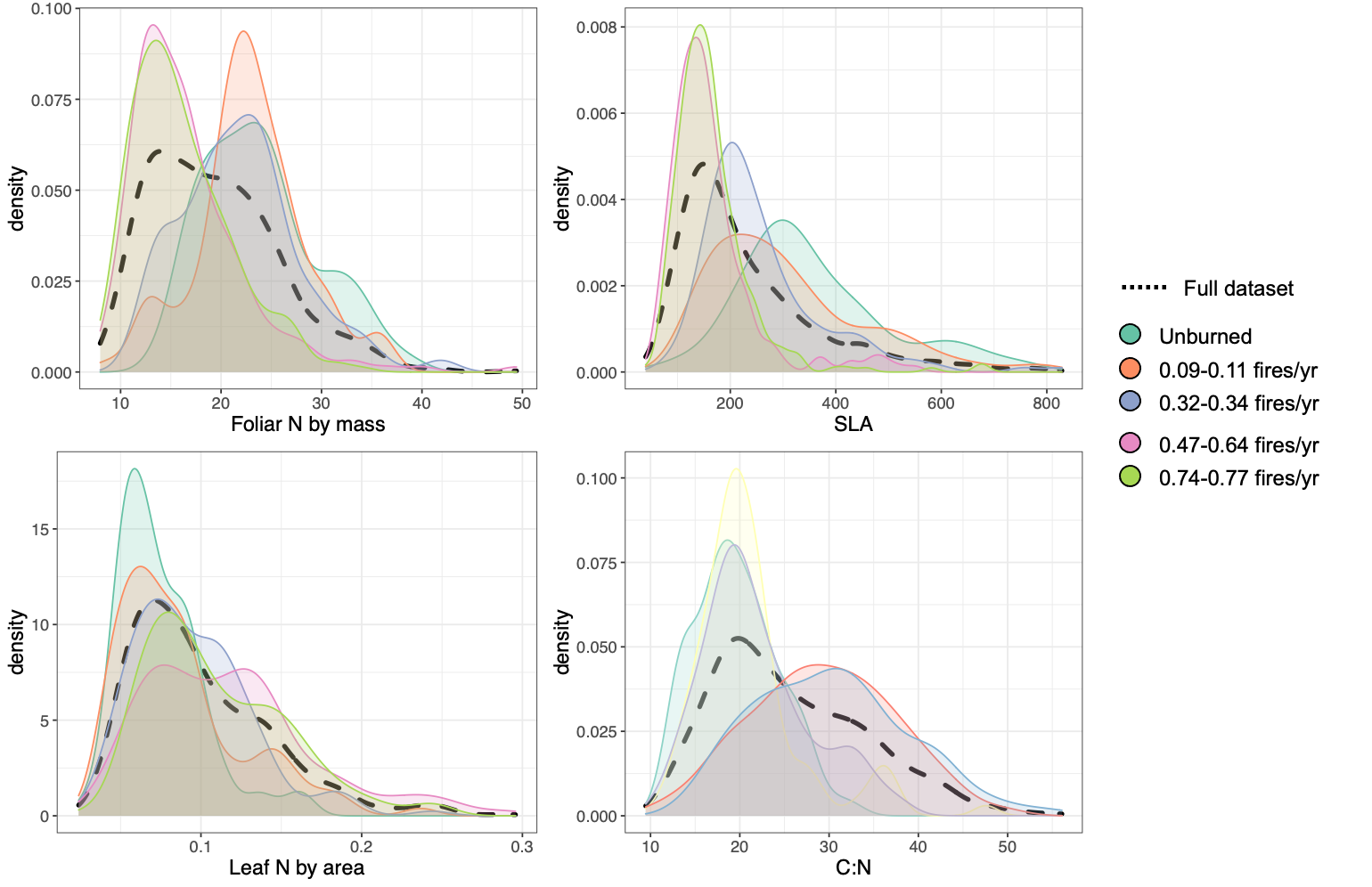


Figure S6: Distribution of traits across fire treatments and the entire trait dataset using density plots. Foliar nitrogen by mass (N, mgN/g) and area (mgN/cm^2^-leaf area), carbon to nitrogen ratio (C:N), and specific leaf area (SLA, cm^2^/g).
